# Effects of a Circuit Training Program on Myokine Levels in Insulin-Resistant Women: A Randomised Controlled Trial

**DOI:** 10.1155/jdr/6624919

**Published:** 2024-11-28

**Authors:** Joanna Karolkiewicz, Monika Krzywicka, Monika Szulińska, Katarzyna Musialik, Dominka Musiałowska, Jacek Zieliński, Agnieszka Bilska, Marzena Ratajczak

**Affiliations:** ^1^Department of Food and Nutrition, Poznan University of Physical Education 61-871, Poznan, Poland; ^2^Department of Cardiological and Rheumatological Rehabilitation, Poznan University of Physical Education 61-871, Poznan, Poland; ^3^Department of Treatment of Obesity, Metabolic Disorders and Clinical Dietetics, Poznan University of Medical Sciences 60-355, Poznan, Poland; ^4^Insulin Resistance Foundation–Healthy Diet and Healthy Life 61-379, Poznan, Poland; ^5^Department of Athletics Strength and Conditioning, Poznan University of Physical Education 61-871, Poznan, Poland; ^6^Department of Medical Biology, Poznan University of Physical Education 61-871, Poznan, Poland

**Keywords:** fibroblast growth factor 21, interleukin-6, interleukin-10, irisin

## Abstract

**Introduction:** Circuit training is a form of body conditioning with endurance and resistance components. Given the function of skeletal muscle as an endocrine organ secreting various myokines involved in maintaining glucose metabolism homeostasis, our study focused on estimating the impact of the implemented training program on the direction of changes in myokines such as interleukin (IL)-6, IL-10, fibroblast growth factor 21 (FGF21), and irisin in women newly diagnosed with insulin resistance.

**Methods:** This prospective controlled trial randomly divided 42 women into two groups. The training group performed circuit training combining strength (50%–80% of one-repetition maximum) and endurance (50%–75% of heart rate reserve) exercises for 3 months, three 33-min sessions weekly. Exercises were performed on five weight and two cardio machines. The control nontraining group did not change their previous activity. Body composition indicators and IL-6, IL-10, FGF21, and irisin levels were measured before and after the intervention. The data for 27 patients were analysed using two-way repeated measures analyses of variance.

**Results:** The pattern of change in serum IL-6 levels over time differed significantly between the groups (*p* < 0.05). The patterns of change did not differ significantly between groups for IL-10, FGF21, and irisin.

**Conclusion:** The circuit training program implemented in women newly diagnosed with insulin resistance significantly increased their serum IL-6 and not their IL-10, FGF21, and irisin levels.

**Trial Registration:** ClinicalTrials.gov: NCT04528693

## 1. Introduction

Exercise training (T) has long been recognized as a vital nonpharmacological therapy for treating insulin resistance (IR) and diabetes [1]. The American College of Sports Medicine has emphasized the importance of structured exercise, backed by substantial evidence, in treating and managing diabetes [2]. Physical exertion stimulates muscles, leading to increased hormonal activity, which involves the release of peptides, microRNAs, and exosome cytokines, collectively known as myokines. Myokines profoundly impact energy metabolism and inflammation, contributing to increased insulin sensitivity, which improves glucose utilisation and regulates glucose and lipid metabolism [3].

Preliminary findings indicate that the myokine profile of primary skeletal muscle cells from patients with Type 2 diabetes mellitus (T2DM) differs from that of insulin-sensitive individuals [4]. However, it is important to note that many myokines also serve as cytokines, some of which are secreted by other tissues and function as inflammatory factors when released into the circulation by immune cells. Consequently, it is challenging to ascertain whether alterations in myokine secretion in plasma/serum are due to systemic inflammation or changes in skeletal muscle secretion [5].

Changes in circulating cytokines play a pivotal mechanistic role in the exercise process countering chronic low-grade inflammation [6, 7]. The enhanced release of anti-inflammatory cytokines from contracting muscles is now widely recognized to amplify the production of anti-inflammatory cytokines in circulating immune cells and inhibit the release of proinflammatory cytokines from adipose tissue [8].

Research indicates that combining aerobic and resistance T may be more beneficial than either training modality alone for treating metabolic disorders [9]. Given the evidence that myokines, induced by a combination of aerobic and resistance T, can reduce IR progression [10], our goal was to assess whether a moderately intensive circuit T program could alter the myokine profile. We aimed to determine whether this alteration could further increase the sensitivity of muscle tissue in women newly diagnosed with IR.

## 2. Materials and Methods

### 2.1. Study Design

This prospective randomised controlled trial was conducted between August 2020 and September 2022. It adhered to the standards of the Declaration of Helsinki and was approved by the Ethics Committee at Poznan University of Medical Sciences in Poland (Ref.: 462/20). It was conducted per the Consolidated Standards of Reporting Trials guidelines for randomised controlled trials.

Forty-two women who met the inclusion criteria were randomly divided into two groups (allocation ratio: 1:2). Randomisation was performed by an independent researcher using the Microsoft Excel RAND function. The T group performed a circuit T program comprising strength exercise interspersed with bouts of endurance exercise conducted on machines integrated with a computer software program (Milon, Germany) for 3 months, three 33-min sessions per week. The control nontraining (NT) group maintained their current physical activity level for 3 months.

Anthropometric and body composition measurements, a one-repetition maximum (1RM) resistance test, and physiological and biochemical indicator analyses related to insulin sensitivity and carbohydrate and lipid metabolism were performed on participants in both groups at the beginning of the study and after the intervention period.

### 2.2. Inclusion and Exclusion Criteria

Participants were recruited through advertisements on the Polish Insulin Resistance Foundation–Healthy Diet and Healthy Life website. Before inclusion, all patients underwent medical examinations at the Department of Treatment of Obesity, Metabolic Disorders and Clinical Dietetics, Poznan University of Medical Sciences. All volunteers were introduced to the study's purpose and protocols and provided written consent to participate.

All study subjects had venous blood samples drawn at the beginning of the study to determine fasting glucose and insulin levels. IR was calculated using the homeostasis model assessment of IR (HOMA-IR) [11]. For participants with fasting plasma glucose levels from 3.0 to 25.0 mmol/L and fasting insulin levels from 3 to 55 mU/mL, glycosylated haemoglobin (HbA1c) was determined to define dysglycemia based on standard HbA1c thresholds [12]. Specifically, T2DM was diagnosed by the presence of one or more of the following criteria: HbA1c of ≥ 6.5%, self-report of T2DM, or use of T2DM medication (oral hypoglycemic agents and/or insulin). Eligible participants were included in this study if they met the following criteria: female, aged 25–45 years, body mass index (BMI) of 25–33 kg/m^2^, HOMA-IR of ≥ 2.0, and no contraindications to physical activity. Patients with any of the following were excluded from this study: menopause, metformin treatment < 3 months before or during the study, Type 1 or 2 diabetes, poorly controlled hypertension (mean systolic blood pressure of > 140 mmHg and/or mean diastolic blood pressure of > 90 mmHg) over the previous month and/or need to modify pharmacological treatment, obesity (BMI > 33 kg/m^2^), blood thyroid − stimulating hormone levels < 0.5 or > 5.0 mIU/L, lipid disorders requiring pharmacological treatment within the 3 months before or during the study, a positive history of ischemic heart disease, carotid and/or lower limb atherosclerosis, clinically significant arrhythmias, conduction disorders, kidney disease, liver dysfunction, acute or chronic clinically manifested inflammatory processes, acute infection in the previous month, dietary supplement use within 1 month before or during the study, taking medications that could interfere with the test results, or other conditions that may pose a risk to the participant during this study.

### 2.3. Intervention

Both study groups were instructed to maintain their usual dietary patterns and the level of physical activity during the study. Dietary intake of energy was verified with 7-day food records at the beginning and end of the 12-week study period, which was previously published [13]. The participants were additionally instructed not to use dietary supplements. The level of physical activity was assessed by the International Physical Activity Questionnaire [14].

**Figure 1:**
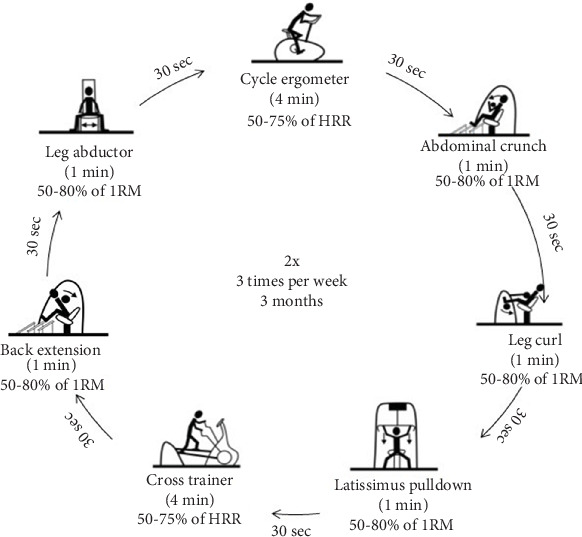
Exercise program.

The T group followed an exercise program comprising circuit T, including strength exercise interspersed with bouts of endurance exercise, performed on the machines shown in Figure 1. All T sessions comprised two exercise series and were conducted on nonconsecutive days for 12 weeks. Each session lasted 33 min. The T program took place at Poznan University of Physical Education. The same team of researchers monitored all sessions. The exercise load was adjusted for each participant using the electronic resistance motor system. The targeted muscle groups were loaded alternately to ensure comprehensive whole-body T. In addition, the actual exercise intensity was monitored during the session on the machines' display monitors.

The designated strength load and intensity were gradually increased during the T program (Table 1) to maintain a moderate intensity level. T intensity was calculated using Karvonen's method [15], and maximum heart rate was calculated by the Milon CARE (Emersacker, Germany) software according to Ferguson's method [16]. The exercise intensity monitoring and 1RM test were performed to determine appropriate T loads and verify patient progression during the program. The 1RM test was performed for each of the five strength exercises included in the circuit. It consisted of a maximum of four repetitions of the exercise with gradually increasing resistance until the participant was not able to overcome the weight. The final fully executed repetition, assessed by the machine's range of motion display and the speed and flow of the movement as observed by the physiotherapist, was considered the 1RM. The applied loads were increased by 1 to 20 kg based on observations from previous repetitions and the patient's rating of perceived exertion.

Procedures were described broadly in previously published article [13].

### 2.4. Measurements

Measurements were performed at the University of Physical Education in Poznań.

#### 2.4.1. Anthropometric and Body Composition Indicators

Anthropometric measurements were performed in the morning, with light clothing and without shoes. Body weight and height were measured using a medical scale with a stadiometer (Seca 285, Hamburg, Germany) to the nearest 0.1 kg and 0.5 cm, respectively. The BMI was calculated based on weight and height using the standard formula. Body composition was assessed using dual-energy x-ray absorptiometry (Lunar Prodigy device; GE Healthcare, Chicago, IL, United States). Total body fat and lean body mass were determined using standard scan mode; the absorbed radiation dose was 0.4 *μ*Gy.

#### 2.4.2. Biochemical Markers

Venous blood samples were collected in the morning after 12 h of fasting and 48 h after the last T session. Blood samples were centrifuged and stored at −80°C until analysis. Serum samples were analysed to measure the glucose and HbA1c concentrations using a Dimension EXL with LM Integrated Chemistry System Analyzer (Siemens, Newark, NJ, United States). Serum insulin was measured using an immunoradiometric assay (DIAsource ImmunoAssays S.A., Nivelles, Belgium) with a manufacturer-reported mean minimum detectable value of 1.0 *μ*IU/mL. *β*-cell function and IR were estimated using the following formulas [11]: *β* − cell function [%] = 20 × fasting insulin [pmol/L]/(fasting glucose [mmol/L] − 3.5) and HOMA − IR index = fasting insulin [mU/L] × fasting glucose [mmol/L]/22.5.

IL-6 and IL-10 were measured using tests from SunRed Biotechnology Company (Shanghai, China). Irisin and FGF21 were measured using enzyme-linked immunosorbent assays from BioVendor Research and Diagnostic Products (Brno, the Czech Republic). The limits of quantitation for IL-6, IL-10, FGF21, and irisin were estimated to be 1.867 pg/mL, 1.142 pg/mL, 7.000 pg/mL, and 1.000 ng/mL, respectively. The intra- and interassay coefficients of variation were < 8% and < 11% for the IL-6 and IL-10 assays, < 2.4% and < 3.5% for the FGF21 assay, and < 8.2% and < 9.7% for the irisin assay, respectively.

### 2.5. Statistical Analysis

Data analyses were performed using the Statistica 13.3 software package (TIBCO Software Inc., Palo Alto, CA, United States). Data are presented as mean ± standard deviation (SD). The normality of each variable's distribution was assessed using the Shapiro–Wilk test. The Pearson chi-squared test and the Student *t*-test were used to assess baseline differences in mean values between T and NT groups. A two-way repeated measures analysis of variance (ANOVA) was used to assess the effects of time, group, and the time × group interaction. Tukey's honestly significant difference post hoc test for unequal *N* was used to assess the significance of differences between pairs of measurements. The coefficient eta squared (*η*^2^) is presented as an effect size indicator. Correlation analysis was conducted using Spearman's rank correlation coefficient. A minimum sample size was determined for the variable we most expected to change (IL-6). The power analysis was performed for repeated measures ANOVA within–between factors in G⁣^∗^Power software (version 3.1.9.4), assuming a large effect size (*η*^2^ = 0.14; ES = 0.403). The correlation coefficient among repeated measures in our pilot study was 0.85. The analysis indicated that a minimum of eight cases per group would be required to provide ≥ 80% power to detect an intervention effect statistically significant at the *α* = 0.05 level.

**Figure 2:**
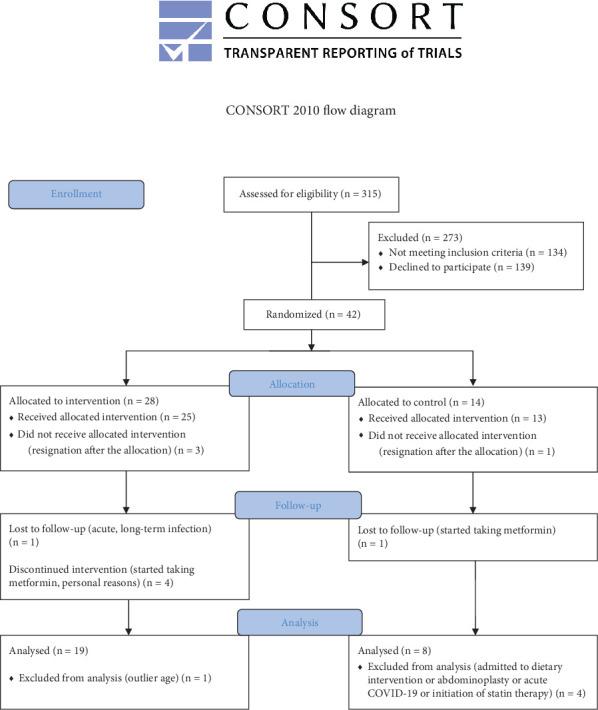
Study flowchart.

The study flowchart is shown in Figure 2. Participants were randomly assigned to the T (*n* = 28) and NT (*n* = 14) groups (allocation ratio: 2:1). Twenty-seven participants completed this study. Attendance at the T program was 96%. Dietary intake did not change during the study, and the level of physical activity increased only in the T group [13].

The demographic and clinical characteristics of women with overweight or mild obesity and IR in T and NT groups are summarized in Table 2. The examined variables did not differ significantly between groups at baseline.

The effects of the circuit T program on body composition are shown in Table 3. The examined variables did not differ significantly between groups at baseline. Repeated measures ANOVA revealed a significant group × time interaction for fat-free mass (*p* < 0.01), indicating a different effect between groups over the 12-week study period. Fat-free mass significantly increased in the T group but did not change in the NT group after 12 weeks. Total body fat tissue, arm fat, and visceral adipose tissue volumes did not differ significantly between the T and NT groups in the same period.

The effects of the circuit T program on the secretion of myokines (IL-6, IL-10, FGF21, and irisin) into the circulation are shown in Table 4. A significant group × time interaction for IL-6 levels was observed over the 12-week study period (*p* < 0.05). The IL-6 concentrations were not homogeneous in the T group, with a relatively high SD, indicating appreciable interindividual variability. IL-10, FGF21, and irisin concentrations did not differ significantly between the T and TN groups over the same period. Repeated measures ANOVA did not reveal significant group × time interactions for IL-10, FGF21, or irisin.

**Figure 3:**
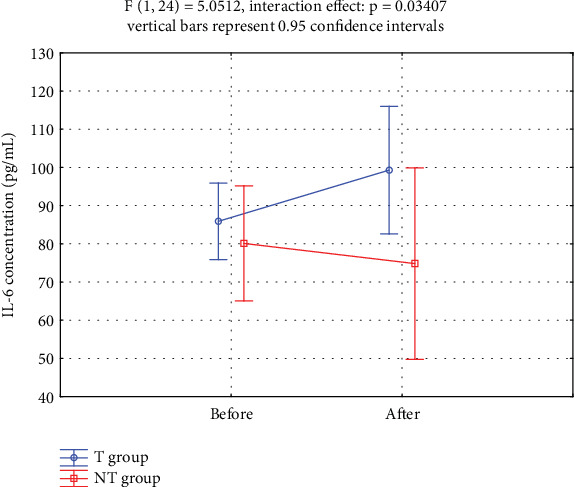
Serum IL-6 concentrations before and after the T intervention in the T and NT groups.

Figure 3 showed that serum IL-6 levels increased significantly in the T group compared to the NT group over the 12-week study period.

**Figure 4:**
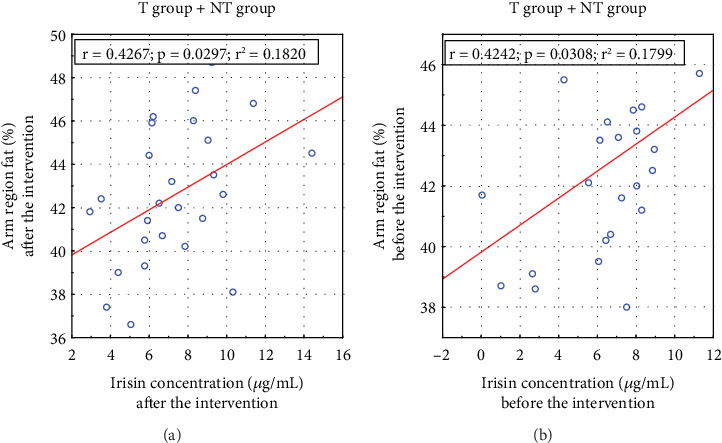
Pearson's correlation between irisin concentrations and arm fat content before (a) and after (b) the intervention.

A positive correlation existed between arm fat content and irisin concentrations before and after the applied T (Figure 4).

## 3. Discussion

Given that physical T modifies the functions of skeletal muscle and adipose tissue in which paracrine regulate glucose metabolism, our study focused on estimating the T program's impact on changes in myokines in women newly diagnosed with IR.

### 3.1. IL-6

When skeletal muscles release high IL-6 concentrations during physical activity due to metabolic stress, it promotes energy mobilisation in adipose and skeletal muscle tissue, enhancing lipolysis and muscle insulin sensitivity [17]. This release also transiently reduces inflammation, making IL-6 an anti-inflammatory molecule, diminishing chronic low-grade inflammation [18, 19].

In our study, IL-6 levels were high in both the T (85.9 ± 23 pg/mL) and NT (80.1 ± 12 pg/mL) groups before the circuit T program (Table 3). Said et al. [20] reported that average IL-6 levels range between 4.63 and 5.74 pg/mL in most healthy individuals. It should be noted that in a rested state, plasma IL-6 levels do not reflect IL-6 release from skeletal muscle but instead reflect production and accumulation from other sources, including leukocytes and adipocytes [21]. The elevated circulating level of IL-6 is an independent predictor of T2DM and is considered to be a contributing factor in the development of inflammation, IR, and *β*-cell dysfunction [22]. Elevated serum IL-6 levels in insulin-resistant states might act to counter alterations in muscle IL-6 signaling, like increased insulin secretion in IR. Febbraio and Pedersen suggested that IL-6 may be elevated in disease states as a consequence rather than a cause of the perturbation in order to downregulate other metabolic dysfunctions [23]. Garneau and Aguer suggest that muscles from patients with T2DM are IL-6 resistant [5].

IL-6 is stimulated by physical activity, but its effect on IR is less clear. During the 12-week supervised circuit T program, serum IL-6 levels significantly increased in the T group compared to the NT group (group × time interaction: *p* = 0.034; Table 3; Figure 3). Given that combined aerobic and resistance exercise was the most effective type of exercise to reduce IL-6 levels in women with overweight and obesity [24], it was intriguing that the circuit T implemented in our study resulted in increased IL-6 levels. A large number of studies have confirmed that regular T decreases basal plasma level of IL-6 and increases basal IL-6 receptor mRNA expression in skeletal muscle resulting in enhanced IL-6 sensitivity [21]. Postexercise changes in the immune system indicators may last for 2 to 24 h. Therefore, we exclude changes in the immune system related to the so-called “open window” as blood collection in our study occurred 48 h after the last T session.

Thus, we hypothesise that higher levels of IL-6 in the T group in relation to the non-T group occurred in response to exercise-induced muscle damage. The greater IL-6 concentrations we see following circuit T may be the result of eccentric muscle contraction which increases the flow of IL-6 into the blood circulation. Jiang et al. [25] have demonstrated that myotubes from T2DM patients are resistant to the acute effect of IL-6 on glucose metabolism. Thus, the increase in IL-6 levels in the T group may be also compensatory and related to the observed lack of change in glucose metabolism [13].

### 3.2. IL-10

The elevation in IL-6 levels during prolonged strenuous exercise is often followed by an increase in the anti-inflammatory cytokines IL-10 and IL-1Ra during the postexercise period [6, 26]. However, the effects of moderate-intensity exercise, which is recommended for the general population, on IL-10 and/or IL-1Ra concentrations appear absent or less pronounced [27–29]. Similarly, in our study, the 12-week T program did not significantly increase IL-10 levels in the T group compared to the NT group (group × time interaction: *p* = 0.418; Table 3). Islam et al. suggested that low circulating IL-10 production in response to an inflammatory stimulus such as exercise was associated with the abnormal metabolic properties of myotubes in T2DM [28].

### 3.3. FGF-21

Under basal conditions, FGF21 is predominantly expressed in the liver and adipose tissue [30]. However, FGF21 expression in and secretion from skeletal muscle increases after exercise [31, 32]. Since FGF21 has been identified as an exercise-responsive factor, we investigated the effect of our circuit T program on its levels in women with IR.

Our study noted high circulating FGF21 levels in both groups before the T program. The average baseline FGF21 concentration was 277 ± 220 pg/mL in the T group and 187 ± 89 pg/mL in the NT group (Table 3), higher than and consistent with the levels typically recorded in healthy individuals (100–200 pg/mL), respectively [33].

The clinical significance of FGF21 in humans remains unclear due to a lack of understanding about the causes and consequences of higher circulating FGF21 levels in metabolic disorders [34]. Several studies have indicated that, while circulating FGF21 levels are elevated, its signal transduction and actions are impaired in the skeletal muscles of patients with T2DM and insulin-resistant human skeletal muscle myotubes with diet-induced obesity [35, 36].

Increasing evidence suggests that elevated FGF21 expression in skeletal muscle stimulated by exercise T can regulate whole-body metabolism. However, the literature on exercise-induced changes in serum/plasma FGF21 levels is inconsistent and contradictory [37]. A systematic review and meta-analysis of chronic-exercise–induced FGF21 changes concluded that exercise alone could induce changes in serum FGF21 levels [38]. The underlying mechanism of exercise-induced effects on FGF21 appears to be the enhanced glucose metabolism in muscle via the upregulation of AMP-activated protein kinase (AMPK) signaling [39].

Studies involving exercise T programs have reported decreased plasma/serum FGF21 levels in individuals with obesity [40, 41] due to attenuated FGF21 resistance [41]. However, no change in circulating FGF21 levels was observed after 8 weeks of endurance T in men with obesity but not T2DM [42] or after 10 weeks of either resistance or aerobic T in women with overweight and T2DM [43].

Similarly, our study found no interaction between group and time for FGF21 in a two-way ANOVA analysis (*p* = 0.3180; Table 3).

### 3.4. IRISIN

Irisin is expressed and secreted by human muscle and adipose tissue in individuals with obesity and IR [44]. Human studies suggest that elevated irisin levels could compensate for the abnormal metabolism and insulin sensitivity of individuals with obesity [45]. Huh et al. demonstrated that higher circulating irisin levels were associated with T2DM development over a 2.6-year study period [46]. Another study showed a positive correlation between circulating irisin levels and IR markers in individuals without T2DM [47] which is in contradiction to meta-analyses where circulating irisin levels are lower in patients with T2DM but not with Type 1 diabetes mellitus compared to healthy subjects [48] or newly diagnosed cases [49].

Our study found that baseline blood irisin levels were 6.6 ± 2.6 ng/mL in trained and 6.1 ± 2.6 ng/mL in nontrained women with newly diagnosed IR (Table 3). Although not universally accepted, currently available reference values for circulating irisin range from 3 to 4 ng/mL [50]. Therefore, the irisin levels we observed in women with IR were slightly higher than those of healthy subjects. Our study participants, all newly diagnosed with IR, had average BMI and HbA1c values of 28.8 ± 3.2 and 28.2 ± 1.5 kg/m^2^ and 5.3% ± 0.31% and 5.2% ± 0.25%, respectively. These results support the hypothesis proposed by Crujeiras et al. that irisin is secreted as an adaptive response to counteract the deleterious effect of excess adiposity on glucose homeostasis [47].

Hwang et al. examined serum irisin levels in 424 individuals, concluding that circulating irisin is dysfunctionally altered in individuals with lower skeletal muscle mass and higher visceral fat [51]. Irisin secretion appears to be a compensatory response to reduced energy expenditure, possibly due to physical inactivity, a high-calorie diet, or innate metabolism defects. Our study found a significant correlation between serum irisin concentrations and arm fat content before (*p* = 0.031) and after (*p* = 0.030) the T intervention (Figure 4). Notably, several studies have proposed the mid–upper arm circumference as a new indicator for predicting IR, potentially replacing other anthropometric measurements [52, 53].

Some studies have identified irisin as a potent inhibitor of inflammatory cytokines, with its production upregulated by physical T [54]. After reviewing data on the physiology and role of irisin in glucose homeostasis, Perakakis et al. suggested that physical T–induced modulations of irisin might be vital to preventing numerous metabolic diseases [55].

Liu et al. found that serum irisin levels correlated positively with physical activity [56]. However, our circuit T program did not affect circulating irisin levels in the participating women (group × time interaction: *p* = 0.592; Table 3). Our findings align with those of other studies that found that circulating irisin levels did not change with physical T in populations with overweight, obesity, metabolic syndrome, or T2DM [57]. After reviewing the role of physical T in modulating circulating irisin, Parada-Sánchez et al. concluded that its increase was only reported in one study using short-term high-intensity T [58] suggesting high intensity might be the critical variable triggering increased irisin secretion from muscles [57]. However, further studies are needed to determine whether irisin promotes insulin sensitivity or whether its levels represent an adaptive response induced by exercise T to counteract impaired glucose homeostasis.

### 3.5. Study Limitations

Our study had three main limitations. First, the effect size was very small for some variables, making it impossible to reject some hypotheses. Second, our results only generalise to similar populations (i.e., young women with IR with BMIs between 24 and 33 kg/m^2^). Third, 10 women in the T group and three in the control group were diagnosed with hypothyroidism. Although, in those women, the level of thyroid hormones was pharmacologically stabilized, the influence of the euthyroid state on the obtained results cannot be excluded. Nevertheless, our study also had some strengths: intergroup homogeneity and control of variables affecting IR, such as diet and the amount of physical activity during the intervention.

## 4. Conclusions

The circuit T program implemented in women newly diagnosed with IR significantly increased their serum IL-6 levels and did not alter their IL-10, FGF21, or irisin levels. Further research is needed to clarify the therapeutic potential of circuit T programs in patients with IR.

## Figures and Tables

**Table 1:** Training plan.

**Resistance training load**		**Endurance training intensity**	
Sessions 1–8	50% of 1RM	Sessions 1–8	50% of HRR
Sessions 9–16	60% of 1RM	Sessions 9–16	60% of HRR
Sessions 16–24	70% of 1RM	Sessions 16–36	75% of HRR
Sessions 25–36	80% of 1RM		

Abbreviation: 1RM, one-repetition maximum; HRR, heart rate reserve.

**Table 2:** Baseline demographic and clinical characteristics of participants in the T and NT groups.

	**T group**	**NT group**	**p** ** value**
	**M** **e** **a** **n** ± **SD** **(n** = 19**)**	**M** **e** **a** **n** ± **SD** **(n** = 8**)**	
Age (years)^†^	33.4 ± 4.5	32.1 ± 5.1	0.534^a^
Body mass (kg)^†^	79.7 ± 9.4	79.4 ± 5.0	0.927^a^
BMI (kg/m^2^)^†^	28.8 ± 3.2	28.2 ± 1.5	0.604^a^
Fat-free mass (kg)	45.5 ± 3.9	47.1 ± 2.8	0.313^a^
Total tissue fat (%)	44.0 ± 3.5	41.9 ± 2.2	0.126^a^
Arm region fat (%)	43.2 ± 2.8	41.1 ± 1.6	0.058^a^
Visceral adipose tissue volume (cm^3^)	1095 ± 521	105 ± 489	0.848^a^
Glucose (mmol/L)	5.3 ± 0.4	5.3 ± 0.3	0.830^a^
Insulin (*μ*U/mL)^†^	13.2 ± 3.8	12.3 ± 5.3	0.276^a^
HOMA-IR^†^	3.1 ± 0.9	2.9 ± 1.1	0.176^a^
HBA1c (%)^†^	5.3 ± 0.3	5.2 ± 0.3	0.418^a^
*β*-cell function (%)	935.6 ± 356.8	878.0 ± 474.8	0.441^a^
Hypothyroidism (*n*)^†^	10	3	0.470^b^

^†^Previously published [13].

^a^Student's *t*-test.

^b^maximum likelihood chi-squared test.

**Table 3:** Pre- and poststudy body composition indicators in the T and NT groups.

	**T group**	**NT group**	**ANOVA**		
	**M** **e** **a** **n** ± **SD** **(n** = 19**)**	**M** **e** **a** **n** ± **SD** **(n** = 8**)**	**Group effect**	**Time effect**	**G** **r** **o** **u** **p** × **time**
			**p** ** value (** **ƞ** ^2^ **)**	**p** ** value (** **ƞ** ^2^ **)**	**p** ** value (** **ƞ** ^2^ **)**
Fat-free mass (kg)					
Pre	45.5 ± 3.9	47.1 ± 2.8	**0.583** (0.01)	**0.750** (< 0.01)	**0.009** ^a^ (0.24)
Post	46.3 ± 3.9	46.4 ± 2.3			
Total tissue fat (%)					
Pre	44.0 ± 3.5	41.9 ± 2.2	**0.229** (0.06)	**0.766** (< 0.01)	**0.076** (0.12)
Post	43.6 ± 3.6	42.5 ± 2.3			
Arm region fat (%)					
Pre	43.2 ± 2.79	41.1 ± 1.6	**0.147** (0.08)	**0.841** (< 0.01)	**0.297** (0.04)
Post	42.9 ± 3.56	41.6 ± 1.7			
Visceral adipose tissue volume (cm^3^)					
Pre	1095 ± 521	1053 ± 489	**0.937** (< 0.01)	**0.786** (< 0.01)	**0.570** (0.01)
Post	1057 ± 496	1066 ± 457			

*Note:* Bolded figures refer to *p* values.

^a^Tukey's post hoc test.

**Table 4:** Pre- and poststudy biochemical indicators in the T and NT groups.

	**T group**	**NT group**	**ANOVA**		
	**M** **e** **a** **n** ± **SD** **(n** = 19**)**	**M** **e** **a** **n** ± **SD** **(n** = 8**)**	**Group effect**	**Time effect**	**G** **r** **o** **u** **p** × **time**
			**p** ** value (** **ƞ** ^2^ **)**	**p** ** value (** **ƞ** ^2^ **)**	**p** ** value (** **ƞ** ^2^ **)**
Glucose (mmol/L)^a^					
Pre	5.3 ± 0.4	5.3 ± 0.3	**0.495** (0.02)	**0.473** (0.04)	**0.629** (0.04)
Post	5.3 ± 0.5	5.1 ± 0.2			
Insulin (*μ*U/mL)^a^					
Pre	13.2 ± 3.8	12.3 ± 5.3	**0.397** (0.02)	**0.532** (0.02)	**0.543** (0.01)
Post	14.7 ± 6.3	12.6 ± 9.0			
HOMA-IR^a^					
Pre	3.1 ± 0.9	2.8 ± 1.1	**0.397** (0.03)	**0.532** (0.02)	**0.543** (0.02)
Post	3.5 ± 1.5	2.9 ± 2.0			
Interleukin-6 (pg/mL)					
Pre	45.5 ± 3.9	47.1 ± 2.8	**0.193** (0.07)	**0.337** (0.04)	**0.034** ^b^ (0.17)
Post	99.3 ± 40.4	74.8 ± 9.5			
Interleukin-10 (pg/mL)					
Pre	4.4 ± 1.2	6.1 ± 3.2	**0.076** (0.12)	**0.396** (0.03)	**0.418** (0.03)
Post	4.3 ± 2.0	5.4 ± 2.1			
Fibroblast growth factor 21 (pg/mL)					
Pre	277.3 ± 221.8	187.3 ± 88.6	**0.476** (0.02)	**0.655** (0.01)	**0.318** (0.04)
Post	256.1 ± 212.3	242.4 ± 107.0			
Irisin (*μ*g/mL)					
Pre	6.6 ± 2.6	6.1 ± 2.6	**0.387** (0.03)	**0.239** (0.06)	**0.592** (0.01)
Post	7.7 ± 2.9	6.5 ± 1.8			

*Note:* Bolded figures refer to *p* values.

^a^Previously published [13].

^b^Tukey's post hoc test.

## Data Availability

The data that support the findings of this study are available from the corresponding author upon reasonable request.

## Nomenclature

1RM: one-repetition maximum
AMPK: AMP-activated protein kinase
BMI: body mass index
FGF21: fibroblast growth factor 21
HbA1c: glycosylated haemoglobin
HOMA-IR: homeostasis model assessment of insulin resistance
HR: heart rate
HRR: heart rate reserve
IL-10: interleukin-10
Il-6: interleukin-6
IR: insulin resistance
NT: nontraining
T: training
T2DM: Type 2 diabetes mellitus
